# Genetic Diversity in Stomatal Density among Soybeans Elucidated Using High-throughput Technique Based on an Algorithm for Object Detection

**DOI:** 10.1038/s41598-019-44127-0

**Published:** 2019-05-20

**Authors:** Kazuma Sakoda, Tomoya Watanabe, Shun Sukemura, Shunzo Kobayashi, Yuichi Nagasaki, Yu Tanaka, Tatsuhiko Shiraiwa

**Affiliations:** 1 Graduate School of Agriculture, Kyoto University, Kitashirakawa Oiwake-cho, Sakyo-ku, Kyoto 606-8502 Japan; 2 Research Fellow of Japan Society for the Promotion of Science, Kyoto, Japan; 3 JST, PRESTO, Kitashirakawa Oiwake-cho, Sakyo-ku, Kyoto 606-8502 Japan

**Keywords:** Natural variation in plants, Photosynthesis, Stomata

## Abstract

The stomatal density (SD) can be a promising target to improve the leaf photosynthesis in soybeans (*Glycine max* (L.) Merr). In a conventional SD evaluation, the counting process of the stomata during a manual operation can be time-consuming. We aimed to develop a high-throughput technique for evaluating the SD and elucidating the variation in the SD among various soybean accessions. The central leaflet of the first trifoliolate was sampled, and microscopic images of the leaflet replica were obtained among 90 soybean accessions. The Single Shot MultiBox Detector, an algorithm for an object detection based on deep learning, was introduced to develop an automatic detector of the stomata in the image. The developed detector successfully recognized the stomata in the microscopic image with high-throughput. Using this technique, the value of R^2^ reached 0.90 when the manually and automatically measured SDs were compared in the 150 images. This technique discovered a variation in SD from 93 ± 3 to 166 ± 4 mm^−2^ among the 90 accessions. Our detector can be a powerful tool for a SD evaluation with a large-scale population in crop species, accelerating the identification of useful alleles related to the SD in future breeding programs.

## Introduction

Crop yield can depend largely on the cumulative rate of photosynthesis throughout the growth period^1^. It is significant to determine the key factor in enhancing the leaf photosynthesis because an increase in leaf photosynthesis can achieve an improvement in the biomass production of crop plants^2^. Gas diffusion through the stomata is known as one of the limiting factors for leaf photosynthesis in plant species. Previous studies demonstrated the positive correlation between the maximum CO_2_ assimilation rate and the stomatal conductance (*g*_s_) under saturated light conditions in several crops^3–5^. The potential of *g*_s_ is mainly determined based on the size, depth, opening of a single stoma, and density^6^. The stomatal density (SD), the number of the stomata per unit of leaf area, was reported to be the main factor responsible for the variation in the potential of *g*_s_ among over 70 soybean accessions^7^. These facts suggest that an increase in SD can result in an enhancement of the leaf photosynthesis in soybeans. For an efficient breeding program to improve the SD, it is therefore important to identify the variation in SD among various soybean accessions, as well as the genetic factors associated with such variation.

Several methods have been developed to evaluate the SD in plant species^8^. With these methods, the number of stomata is counted using microscopic images of the intact or peeled epidermis of a leaf, as well as a replica of the leaf surface. Because it is typically applied manually, the counting process of the stomata can be time-consuming, and easily generate errors during the observations. Thus, a new technique to automate this process is needed to evaluate the SD in a large-scale soybean population with high accuracy and efficiency. The deep learning approach, particularly a convolutional neural network (CNN), has become a powerful tool for image analysis in recent years. Various algorithms for object detection have been developed based on a CNN, which has resulted in a remarkable improvement in the accuracy of object detection^9–13^. Some of these algorithms have already been introduced in the detection of pests or diseases in images for several different plant species^14^. Therefore, these algorithms can be considered capable of detecting the stomata in microscopic images. The Single Shot MultiBox Detector (SSD) is a notable algorithm for the detection of multiple objects, which suggests that it can be suitable for the development of an automatic stoma detector^15^.

In the present study, we cultivated various soybean accessions with genome-wide genetic diversity under green house and field conditions. The SSD was introduced to develop an automatic stoma detector for use with microscopic images. A new detector realized a high-throughput evaluation of the SD, and the genetic diversity in the SD was elucidated among various soybean accessions.

## Results

### Accuracy of the developed technique in evaluating the stomatal density based on the SSD algorithm

**Figure 1:**
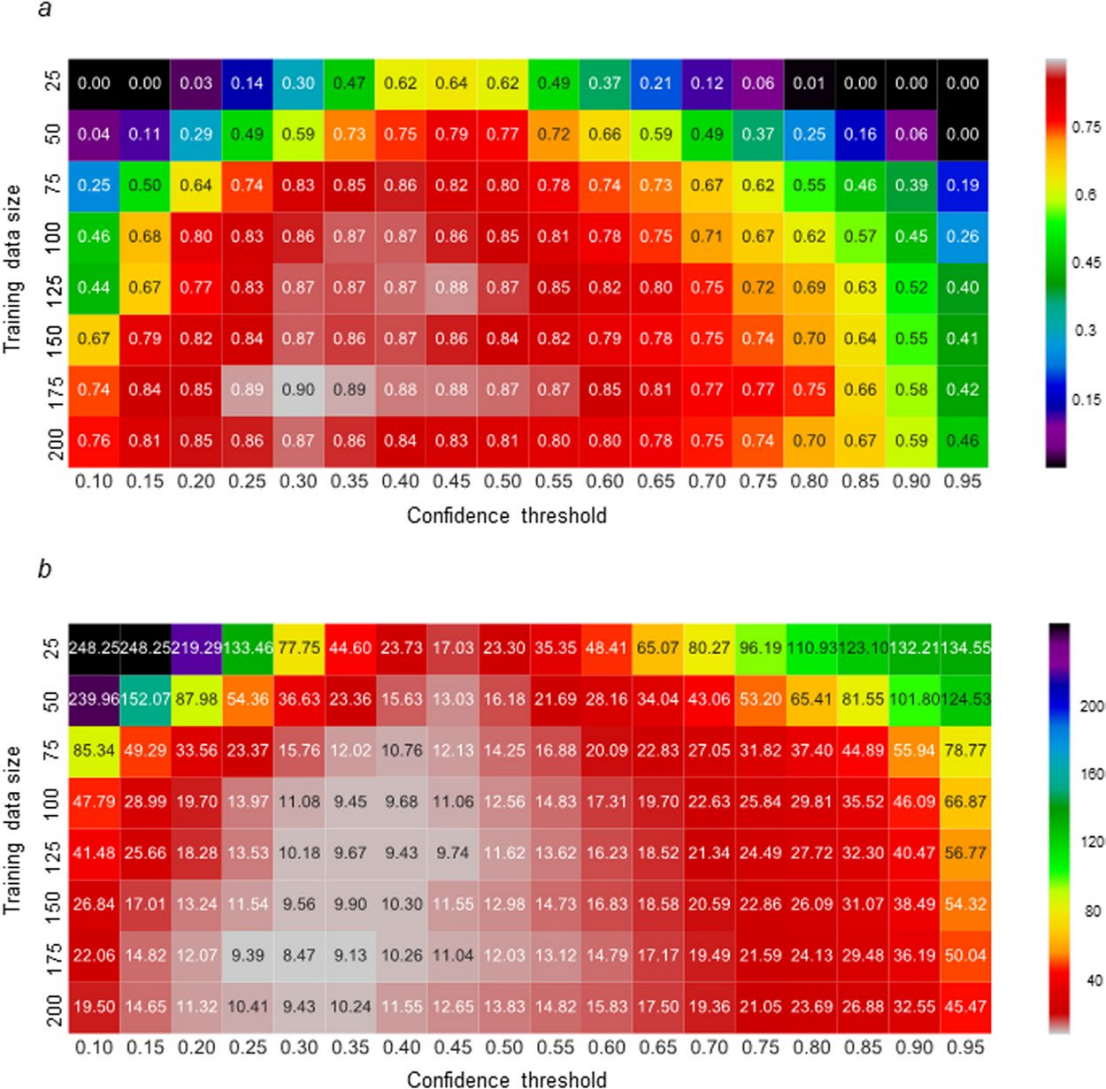
Comparison of the output accuracy using the models trained with different dataset sizes and various confidence thresholds of the stomata. The output accuracy was evaluated based on (**a**) the coefficient determination (R^2^) and (**b**) RMSE when the manually and automatically measured SDs were compared in the 150 images as the test datasets. The SD was measured in the test dataset using the models trained with 25, 50, 75, 10, 125, 150, 175, and 200 images, and a confidence threshold of the stomata of 0.1 to 0.95 at intervals of 0.05. The color scales indicate the degree of the output accuracy (white, higher accuracy; black, lower accuracy).

**Figure 2:**
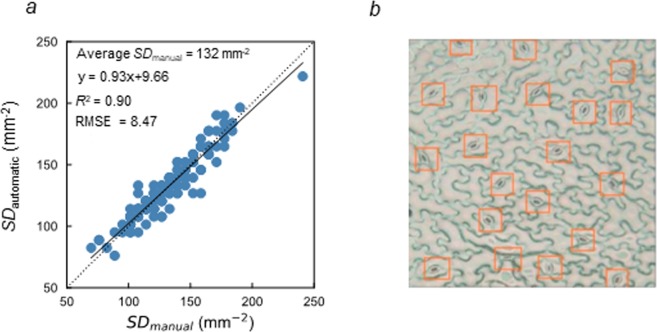
The relationship between the manually and automatically measured stomatal density using the optimum model and confidence threshold of the stomata. (**a**) Combining the optimum model and confidence threshold of the stomata, R^2^ was 0.90 and RMSE was 8.47 when the manually and automatically measured SDs were compared in the 150 images as the test dataset. (**b**) Example of the input image using the developed technique were selected from the test dataset. In (**b**), the detected stomata are enclosed with orange boxes. The liner regression equations applied can be described as follows: y = 0.93x + 9.66 in (**a**).

In the present study, we compared the output accuracy using the models trained based on the different sizes of the training dataset and the various values of *C*_st_. The output accuracy was evaluated based on the R^2^ and RMSE values calculated from the relationship between the manually and automatically measured SD in the test datasets. The model trained within a dataset size of 125 to 200 scored the highest R^2^ and lowest RMSE values with each *C*_st_ (Fig. 1a,b). In addition, similar results in the R^2^ and RMSE values were shown with each model, when *C*_st_ was set from 0.30 to 0.45. There was no significant variation in the R^2^ and RMSE values when the trained model with 100 to 200 images and the *C*_st_ of 0.30 were applied to 10 batches of 50 images sampled from the test dataset (Supplementary Fig. S1). These results suggest that the optimum sizes of the training dataset and *C*_st_ can be fixed at within 125 to 200, and 0.3 to 0.45, respectively. The value of R^2^ was the highest (0.90), and the RMSE value was the lowest (8.47), when the model was trained using 175 images and *C*_st_ was set to 0.30 (Fig. 2a). The average *SD*_manual_ was 132 mm^−2^ and the lowest RMSE value was 6.4% of the average *SD*_manual_. These results confirm that the optimum model and *C*_st_ used in the present study can be useful for the automatic detection of the stomata in an image. Example of the output image using the optimum model and *C*_st_ are shown in Fig. 2b.

### Versatility and expansibility of the developed technique used to evaluate the stomatal density

**Figure 3:**
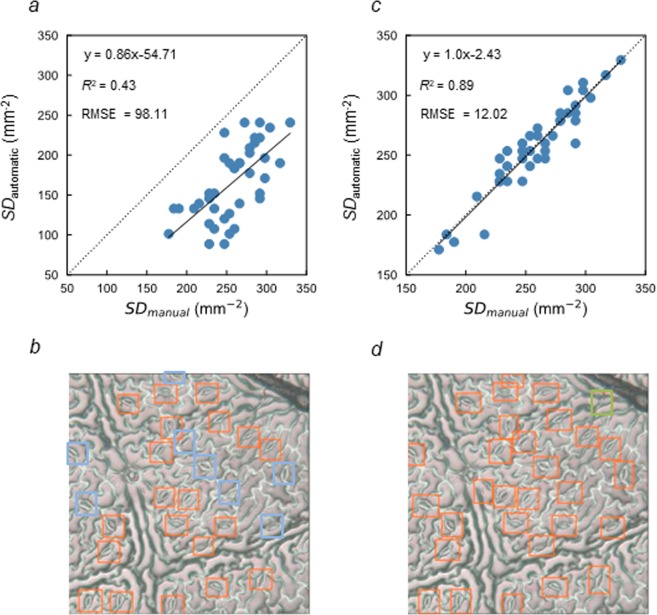
Versatility and expansibility of the optimum model and confidence threshold of the stomata. (**a**) Combining the optimum model and confidence threshold of the stomata (*C*_st_) developed during the greenhouse experiment, the R^2^ was 0.43 and RMSE was 98.1 when the manually and automatically measured SDs were compared in the 40 images obtained during the field experiment. (**b**) From the 40 images, the example of the output image using the developed technique were selected. (**c**) Combining the fine-tuned model and the *C*_st_ of 0.45, the R^2^ was 0.89 and RMSE was 12.02 when the manually and automatically measured SDs were compared in the 40 images obtained during the field experiment. (**d**) From the 40 images, the example of the output image using the fine-tuned model and the *C*_st_ of 0.45 were selected. In (**b**,**d)**, the detected stomata are enclosed with orange boxes, whereas the non-detected and mis-detected stomata are enclosed with blue and green boxes, respectively. The liner regression equations applied can be described as follows: y = 0.86x −54.71 in (**a**) and y = 1.00x −2.43 in (**c**).

To test the versatility of the optimum model and *C*_st_ developed through the greenhouse experiment, they were applied to the 40 images obtained from the soybean accessions after the flowering stage and under field conditions. The R^2^ and RMSE values were 0.43 and 98.11, respectively, when the manually and automatically measured SD were compared (Fig. 3a). Example of the output image using the optimum model and *C*_st_ are shown in Fig. 3b.

To test the expansibility of the optimum model developed through the greenhouse experiment, the fine tuning was conducted to that optimum model as the basal model with 45 images. It was applied to the 40 images for the versatility test with the combination of various C. The fine-tuned model scored the highest value of R^2^ (0.89) and the lowest value of RMSE (12.02) with the *C*_st_ of 0.45 (Fig. 3c). Example of the output image using the fine-tuned model are shown in Fig. 3d.

### Genetic diversity in the stomatal density and leaf area among soybeans

**Figure 4:**
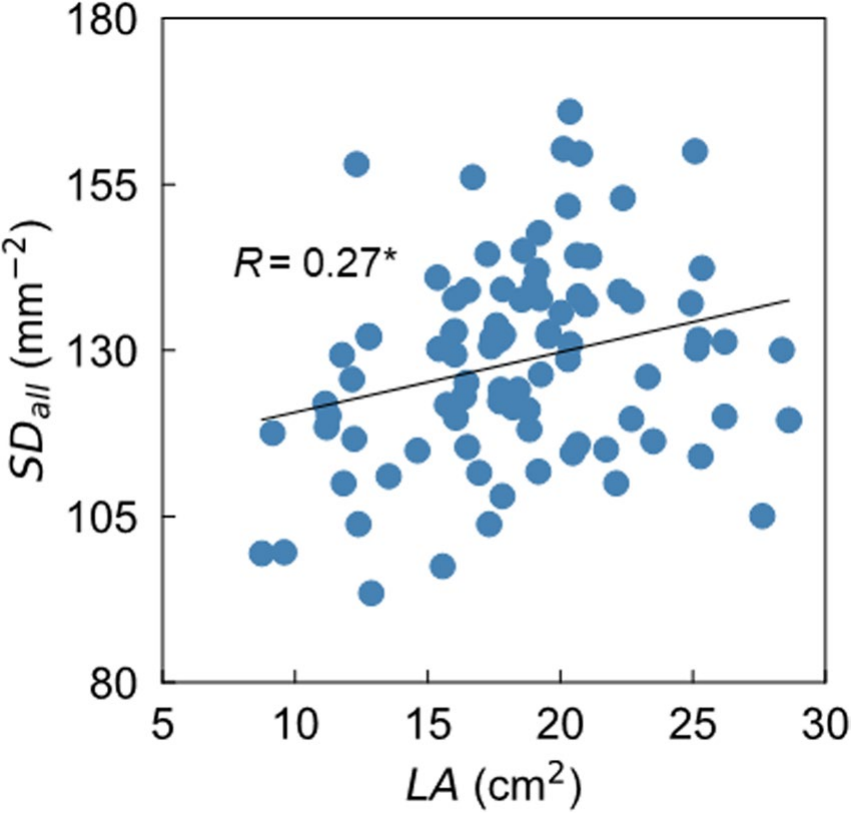
Relationship between the stomatal density and leaf area among the 90 soybean accessions. The relationship between the stomatal density in a whole single leaf (*SD*_all_) and the LA was investigated among the 90 soybean accessions. The solid line indicates the liner regression between *SD*_all_ and LA with a coefficient correlation (*R*) of 0.27. Here, *indicates that the correlation between *SD*_all_ and LA was significant at *p* < 0.05, according to the Pearson’s product moment.

In the greenhouse experiment, we evaluated the SD on the leaflet of the first trifoliolate in the 90 soybean accessions using the model trained using 175 images and a *C*_st_ of 0.30. Among the accessions, this technique discovered a variation in *SD*_base_ from 94 ± 5 to 162 ± 6 mm^−2^, *SD*_middle_ from 87 ± 7 to 168 ± 19 mm^−2^, *SD*_tip_ from 91 ± 7 to 177 ± 15 mm^−2^, and *SD*_all_ from 93 ± 3 to 166 ± 4 mm^−2^ (Table 1). The mean values of each SD in the 90 accessions were 127 ± 4, 126 ± 4, 132 ± 4, and 128 ± 2 mm^−2^, respectively. The accession [*a*] and position [*p*] had significant effects on the SD (*p* < 0.01), whereas there was no the significant effect from the interaction between [*a*] and [*p*]. Among the 90 accessions, the value of LA varied from 8.74 ± 0.54 to 28.62 ± 4.62 cm^2^, which was significant (*p* < 0.01). The mean value of LA in the 90 accessions was 18.38 ± 0.47 cm^2^. There was a positive and significant correlation between *SD*_all_ and LA (*R* = 0.27, *p* < 0.05) (Fig. 4).

**Table 1:** Variation in stomatal density and leaf area among 90 soybean accessions.

	Stomatal density (mm^−2^)			Leaf area (cm^2^)		
position	min	max	mean	min	max	mean
*SD*_base_	94 ± 5	162 ± 6	127 ± 4	8.74 ± 0.54	28.62 ± 4.62	18.38 ± 0.47
*SD*_middle_	87 ± 7	168 ± 19	126 ± 4			
*SD*_tip_	91 ± 7	177 ± 15	132 ± 4			
*SD*_all_	93 ± 3	166 ± 4	128 ± 2			
accession [*a*]	**			**		
position [*p*]	**					
[*a*] × [*p*]	n.s.					

The SD and LA were evaluated in a central leaflet at the first trifoliolate in 90 soybean accessions (n = 3–4). The SD was evaluated at the basal, middle, and tip positions on the abaxial side of the single leaflet, as indicated by *SD*_base_, *SD*_middle_, and *SD*_tip_, respectively. The mean value of SD for the three positions was calculated as *SD*_all_. Each value of the SD and LA indicate the mean followed by a standard error. Here, **indicates the significant effect of the accession [*a*] and position [*p*] on the SD and LA at *p* < 0.01, according to a one-way or two-way ANOVA. In addition, n.s. indicates that the interaction of [*a*] and [*p*] on the SD was not significant.

**Figure 5:**
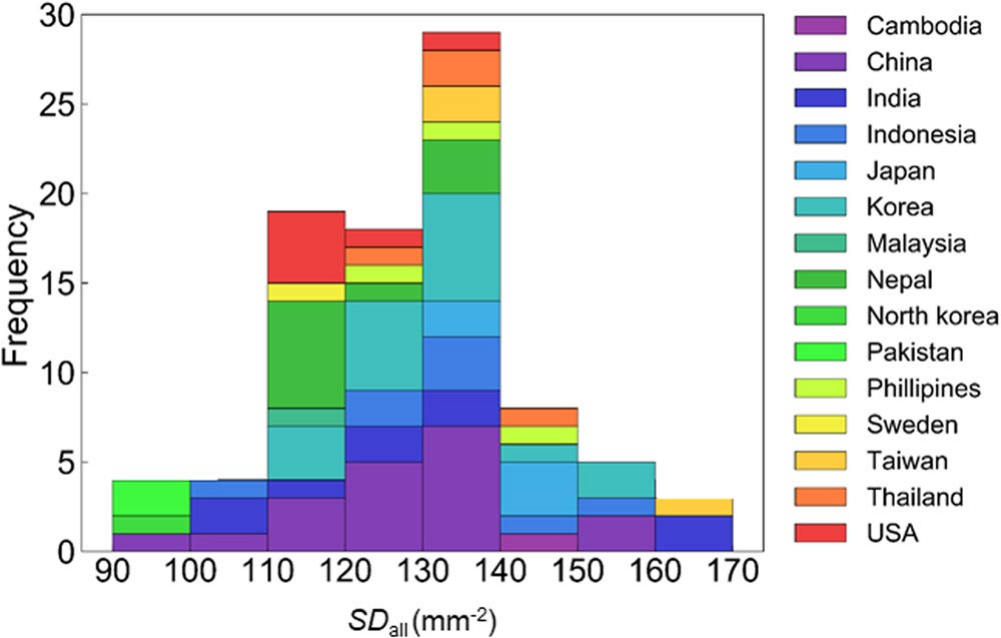
The distribution of SD among the 90 soybean accessions. The distribution of the SD in an entire single leaf (*SD*_all_) among the 90 soybean accessions was described as a histogram. The 90 accessions were classified in 15 groups based on the origin. The histogram was generated by stacking the distribution of the 15 groups with each specific color.

The distribution of *SD*_all_ among the 90 accessions was described using a histogram with 15 different colors classified based on the origin of the accessions (Fig. 5). “Pochal,” an accession from Taiwan, showed the highest *SD*_all_ (166 ± 4 mm^−2^) of the 90 accessions, whereas “Masshokutou,” an accession from China, showed the lowest *SD*_all_ (93 ± 3 mm^−2^). There was no significant variation in *SD*_all_ among the accession groups classified based on their origins, whereas there was the large variation in *SD*_all_ among the accessions within each group.

## Discussion

In the present study, we aimed to develop a novel technique to evaluate the SD with high efficiency in soybean. ImageJ, an open source image processing program, is widely known to be useful for the image analysis^16^. It has been used for the cell counting in the manual and automatic methods in the previous studies^7,17^. However, the tools for object detection in ImageJ could not be applicable for the automatic detection of the stomata in the images generated by SUMP since the structure of the stomata is highly similar to that of the ruggedness on leaf surface. The deep learning approach has been recently introduced into crop phenotyping^14,18,19^. To the best of our knowledge, the present study is the first report utilizing deep learning to evaluate the SD in crop species. It was confirmed that the new detector developed in the present study recognized the stomata in the microscopic images with high accuracy, at least during the specific growth stage of soybean (Figs 1 and 2).

The automatic technique needs to be accurate enough to elucidate the genetic diversity in the SD among soybean accessions. Combining the optimum model and the *C*st, the average SD was 132, while the RMSE value was 8.47 in 150 images as the test dataset (Fig. 2). In the previous studies, the variation in the SD ranged 42.6% and 74.0% among 43^20^ and 77^7^ soybean accessions, respectively. The RMSE value was only 6.4% of the average SD, and this percentage was much smaller than the variation range in the SD among soybeans shown in the previous studies. It implies that the developed model has achieved the acceptable accuracy to elucidate the genetic diversity in the SD among soybeans. This technique automates the counting process of the stomata in an image, that is, it can archive a higher SD evaluation efficiency than a manual method. It is expected to be a powerful tool for an SD evaluation in a large-scale soybean population, which can accelerate the phenotyping in a genetic analysis. The approach utilized here can also be introduced to an SD evaluation in other plant species. Therefore, the present study has the potential to accelerate the identification of useful alleles related to the SD, which might contribute to an enhancement of the leaf photosynthesis throughout the crop breeding.

We developed an automatic detector of stomata using the image dataset obtained from the soybean plants during the early growth stage and under controlled conditions. To obtain the optimum model and the *C*_st_, we conducted the training using the dataset of different sizes and the SD was measured using each model with the combination of various *C*_st_. The highest accuracy was shown using the model trained by 175 or 200 images with all the *C*_st_ except for 0.4 and 0.45 (Fig. 1). In addition, the R^2^ and RMSE values were calculated from the relationship between the manually and automatically measured SD using all the trained models and the *C*_st_ of 0.3 in 10 batches of 50 images sampled from the test dataset, respectively. The significant increase in the output accuracy was shown using the trained models with the larger dataset size within 25 to 100 images (Supplementary Fig. S1). On the other hand, there was no significant variation in the output accuracy using the trained models with 100 to 200 images. These results suggest that the training dataset size of 100 images can be large enough to obtain the applicable model for the stomata detection based on the SSD algorithm in the present study.

To test the versatility of the developed detector, it was applied to the dataset obtained from the plants during the later growth stage and under field conditions. The output accuracy was lower in the dataset obtained during the field experiment (R^2^ = 0.43, RMSE value = 98.1) than that obtained during the greenhouse experiment (R^2^ = 0.90, RMSE value = 8.47) (Figs 2a and 3a). The difference in the growth stage and environmental conditions can cause a difference in the morphological characteristics of the leaflet, such as the number, size, or shape of the stomata and epidermal cells in soybeans. This might be responsible for the difference in the output accuracy between the image datasets. These results indicate that different models and *C*_st_ should be developed when the morphological characteristics are expected to vary largely among the leaflet samples. On the other hand, the output accuracy using the fine-tuned model and the *C*_st_ of 0.45 was comparable (R^2^ = 0.89, RMSE value = 12.0.) (Fig. 3c) with that obtained during the greenhouse experiment. It should be noted that the dataset size of only 45 images was large enough to develop this fine-tuned model. These results confirm that the fine tuning is an effective approach to develop the adapted model for each case. The developed detector during the greenhouse experiment can be utilized as the basal model for the fine tuning with the training dataset of a small size.

In the present study, we introduced SUMP protocol, which is simple and efficient method for the stomata observation. The nail polish impression method^21^ and cuticle preparation method^8^ are widely applied to the stomatal observation. It is common between the SUMP and nail polish impression method to prepare and observe the replica of leaf surface. It is expected that the automatic technique developed in the present study can be applied to the images obtained by both methods with the comparable accuracy. On the other hand, the image quality using cuticle preparation method would be largely different from those using the SUMP or the nail polish impression method because the stomata are directly observed on leaf surface. Cuticle preparation method has the limitations for the stomata observation as it would be suitable for the leaf whose epidermis can be easily separated and it would be time-consuming for the preparation. Thus, cuticle preparation method can be introduced to the SD evaluation using the automatic technique by the application of fine-tuning technique and the improvement of the preparation adoptability and efficiency.

There have been a few reports showing the variation in SD among the different positions in a single leaf in different plant species. In the present study, the position in a single leaflet had a significant effect on the SD (Table 1). The SD at the tip position was higher than that at the basal and middle positions. These results suggest that the distribution of the stomata can differ among the positions in a single soybean leaflet. It seems to be favorable for researchers to evaluate the SD for a similar position through a comparison among different leaf samples.

The positive correlation between the SD and LA was shown in a woody plant^22^. In the present study, a positive correlation between *SD*_all_ and LA was also shown, although the correlation coefficient was small (R = 0.27, *p* < 0.05) (Fig. 4). In contrast, Tanaka *et al*.^7^ showed a negative correlation between them among 77 soybean accessions, mostly consisting of Japanese and US cultivars (R = 0.43). These results indicate that the relationship between the SD and LA can be inconsistent across the soybean accessions, and that the variation in LA in soybeans might not be closely related to the variation in SD.

The previous studies showed a variation in SD from 242 to 345 mm^−2^ among 43 soybean accessions^20^, and from 192 to 334 mm^−2^ among 77 soybean accessions^7^, after the flowering stage. In the present study, we discovered a significant variation in *SD*_all_ of 93 ± 3 to 166 ± 4 mm^−2^ among the 90 soybean accessions (Fig. 5). This indicates that a genetic variation in SD can be detected among the soybean accessions during the early growth stage. Tanaka *et al*.^7^ showed a significant variation in SD between groups consisting of Japanese or US soybean cultivars. In contrast, there was no clear variation in SD among the accession groups classified based on their origin, although in the present study there was a large variation among the accessions within each group. It is therefore suggested that the different genetic factors can be associated with a variation in SD among the accessions at the different growth stages of soybean.

In conclusion, we succeeded in the development of a high-throughput technique for an SD evaluation based on an algorithm for object detection in soybeans. This technique shows a significant variation in SD among 90 soybean accessions with genome-wide genetic diversity, and can be a powerful tool for an SD evaluation with large-scale populations in crop species, which can accelerate the identification of useful alleles related to SD in future breeding programs.

## Methods

### Plant materials and cultivation

We conducted an experiment in a greenhouse using 90 soybean accessions consisting of 78 accessions from the World Soybean Core Collection, which was developed by the National Institute of Agrobiological Sciences (NIAS)^23^, and 12 accessions originating from Asian countries and the USA^24^ (Supplementary Table S1). The NIAS core collection was designed to have genome-wide genetic diversity based on genotype information presented by the SNPs markers. One plant was sown in a vinyl pot with a diameter of 9 cm, containing a mixture of equal amounts of vermiculite and granular culture soil. Four pots were prepared for each accession. The sowing date was November 13, 2017. The plants were randomly placed and grown in a greenhouse located at Kyoto University, Kyoto, Japan (Lat. 35°2′N, Long. 135°47′E, and 65 m altitude). The room temperature was set to 26 °C/20 °C for 14/10 h. All plants were equally watered and fertilized using a liquid fertilizer as needed.

We conducted the field experiment for two years using Enrei and Peking plants, which were included in the 90 accessions used in the greenhouse experiment, and 10 accessions derived from a cross between Enrei and Peking plants, which were developed by Watanabe *et al*.^25^. The experiment was conducted at the Experimental Field of the Graduate School of Agriculture, Kyoto University, Kyoto, Japan (Lat. 35°2′N, Long. 135°47′E, and 65 m altitude, Fulvic Endoaquepts soil type). The sowing date was June 27, 2017 and June 26, 2018. The spacing between rows and plants was 0.7 and 0.15 m, respectively. The N, P_2_O_5_, and K_2_O fertilizers were applied at 3, 10, and 10 g m^−2^, respectively, prior to sowing. Two experimental replications were established for each accession, and each replication was composed of eight plants in a single row in 2017. In 2018, three experimental replications were established for each line, and each replication was composed of 40 plants in four rows.

### Analysis of the leaf area and stomatal density

In the greenhouse experiment, the central leaflets on the first trifoliolate were sampled from all accessions on days 28 and 33 after planting, after each leaflet was fully expanded. The scanned images of the leaflets were obtained using a scanner to measure the leaf area (LA), and the image-processing program, ImageJ (NIH, Bethesda, MD, USA), was applied. To evaluate the SD, the replicas on the abaxial side of the leaflet were prepared at the basal, middle, and tip positions, respectively, for each leaflet, using Suzuki’s Universal Method of Printing (Supplementary Fig. S2). These replicas were observed at a 100X magnification using an optical microscope, and three microscopic images were obtained for each replica (CX31 and DP21, Olympus, Tokyo, Japan). In total, 3216 microscopic images of the leaflet replica were obtained in the greenhouse experiment.

In the field experiment, the fully expanded and central leaflets on the upper-most trifoliolate were sampled from the six accessions on day 53 after planting in 2017 when the accessions mostly reached the beginning of the seed filling and from the eight accessions on day 37 and 49 after planting in 2018 when the accessions mostly reached the beginning of the flowering and the seed filling, respectively. One replica was prepared at the middle position in each leaflet, and 6–9 microscopic images were obtained for each accession using the same method as applied in the greenhouse experiment.

The SD was evaluated in all images using the technique developed in the present study. In the greenhouse experiment, the mean values of the SD in three images obtained at each position were calculated as *SD*_base_, *SD*_middle_, and *SD*_tip_, respectively (n = 3–4). The mean value of *SD*_base_, *SD*_middle_, and *SD*_tip_ was also calculated as *SD*_all_ in each leaflet (n = 3–4).

### Development of high-throughput technique to evaluate the stomatal density based on an algorithm for object detection

In the present study, the SSD, an algorithm for object detection based on deep learning, was introduced to develop an automatic stoma detector for use with microscopic images. The SSD has a network architecture based on VGG16, one of the standard architectures for a CNN, and was designed to be end-to-end trainable. The SSD was shown to detect multiple objects in an image with high accuracy. This suggests that the SSD can be a suitable algorithm for the detection of multiple stomata in an image.

We originally obtained microscopic images with a pixel resolution of 1,840 × 1,840 from leaflet replicas, and then resized them to a pixel resolution of 300 × 300 prior to the training and output processes. Two hundred images from the 3,216 images obtained during the greenhouse experiment were randomly sampled as the training dataset. In the images used as the training dataset, the locations of the stomata were annotated as the ground truth box, using the graphical image annotation tool, LabelImg (Tzutalin. LabelImg. Git code, 2015) (Supplementary Fig. S3). The data regarding the annotations of the stomata in the training dataset were saved as XML files, which were converted into a pickle file to conduct deep learning based on the SSD algorithm. Another 150 images were also randomly sampled from 3,216 images as the test dataset, in which the SD was manually measured.

In the SSD algorithm, the object in the image is classified based on the probability, and can subsequently be detected if the probability is higher than the confidence threshold (C), which should be set to a given value of more than zero for each class. The set value of C can affect the output accuracy for object detection using the SSD algorithm. Moreover, the size of the training dataset can have a certain impact on the output accuracy of the model developed through deep learning. Considering these facts, we conducted training using 25, 50, 75, 100, 125, 150, 175, and 200 images in the training dataset. The epoch size was 50 for all training conducted. Using these models, the SD was measured by changing C of the stomata (*C*_st_) from 0.10 to 0.95 at intervals of 0.05. This allowed us to ensure how many images for the training were used, and which value of C is optimum to detect the stomata in an image. The output accuracy was evaluated based on the R^2^ and RMSE values calculated from the relationship between the manually and automatically measured SD in the test datasets. The trained model and *C*_st_, with which the highest R^2^ and lowest RMSE values were shown, were combined to measure the SD for the 3,216 images in the greenhouse experiment. Subsequently, 10 batches of 50 images were randomly sampled from 150 images as the test dataset. The R^2^ and RMSE values were calculated the from the relationship between the manually and automatically measured SD using all the trained models and the *C*_st_ of 0.3 in each batch, respectively. Tukey-Kramer test was applied to evaluate the significance of the variation in the R^2^ and RMSE values calculated by using the different models.

To test the versatility of the optimum model and *C*_st_ developed through the greenhouse experiment, they were both applied to the 40 images as the test dataset obtained in the field experiment. The R^2^ and RMSE values were calculated from the relationship between the manually and automatically measured SD. In addition, we conducted the fine tuning to test the expansibility of the optimum model developed through the greenhouse experiment. The fine tuning is the technique to obtain the newly adjusted model by the training with the pre-trained model. In the present study, the optimum model developed through the green house experiment was used as the basal model and re-trained with the 45 images obtained in the field experiment other than the test dataset for the versatility test. The fine-tuned model was applied to the 40 images for the versatility test by changing the *C*_st_ from 0.1 to 0.95 at intervals of 0.05. The R^2^ and RMSE values were calculated by the same method with the versatility test.

We obtained the codes to operate the SSD algorithm using the Keras framework on Python (Python Software Foundation, Delaware, USA) from the following location: https://github.com/rykov8/ssd_keras. We modified them to develop an automatic stoma detector for use with microscopic images. The datasets and codes generated and analyzed during the present study are available from the corresponding author on a reasonable request.

### Statistical analysis

A two-way analysis of variance (ANOVA) was applied to evaluate the effects of the accession [*a*], position [*p*], and interaction between [*a*] and [*p*] on the SD. The variation in LA among the accessions was evaluated through a one-way ANOVA. Tukey’s multiple comparison was applied to compare the variation in *SD*_all_ among the accession groups classified by their origins. The significance of the correlation between *SD*_all_ and LA was evaluated according to the Pearson’s product moment correlation coefficient. In the present study, all statistical analyses were conducted using R software (R Foundation for Statistical Computing, Vienna, Austria.).

## Supplementary information

Supplementary Information

## Data Availability

The datasets and codes generated and analyzed during the present study are available from the corresponding author on a reasonable request.
