# Supplementary material for: Genetic Diversity in Stomatal Density among Soybeans Elucidated Using High-throughput Technique Based on an Algorithm for Object Detection
Source: Sci Rep. 2019 May 20;9:7610. doi: 10.1038/s41598-019-44127-0 (PMC6527681; doi:10.1038/s41598-019-44127-0)
Supplement: Supplementary file 1 — Supplementary Information [file 41598_2019_44127_MOESM1_ESM.pdf]

Genetic Diversity in Stomatal Density among Soybeans Elucidated Using High-throughput Technique Based on an Algorithm for Object Detection

Kazuma Sakoda, Tomoya Watanabe, Shun Sukemura, Shunzo Kobayashi, Yuichi Nagasaki, Yu Tanaka, and Tatsuhiko Shiraiwa

Fig. S1 Comparison of the output accuracy using the trained models with the different size of the dataset.

The (*a*) R^2 and (*b*) RMSE values were calculated from the relationship between the manually and automatically measured SD using the trained models with 25, 50, 75, 100, 125, 150, 175 and 200 images and the confidence threshold of the stomata of 0.3 in 10 batches of 50 images sampled from the test dataset, respectively. Tukey-Kramer test was applied to evaluate the significance of the variation in the R^2 and RMSE values calculated by using the different models. Different letters on each column mean the significant variation (p<0.05).

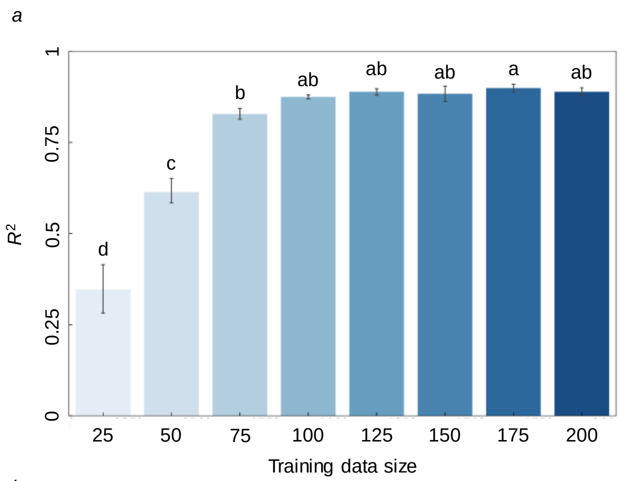

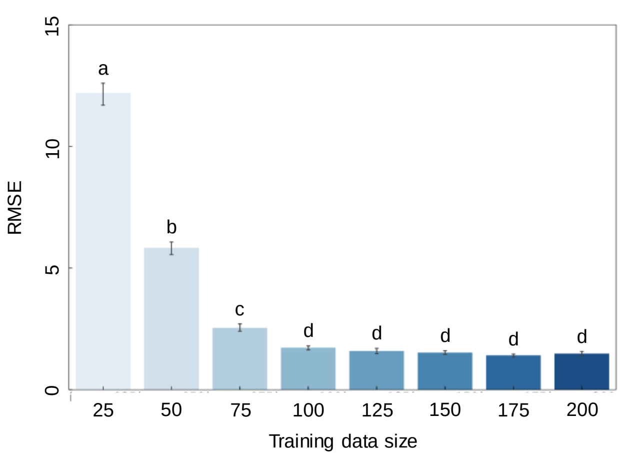

Fig. S2 Preparation method for the replicas at three positions on the abaxial side of the leaflet.

The replicas on the abaxial side of the single leaflet were prepared at the basal, middle, and tip positions using Suzuki's Universal Method of Printing.

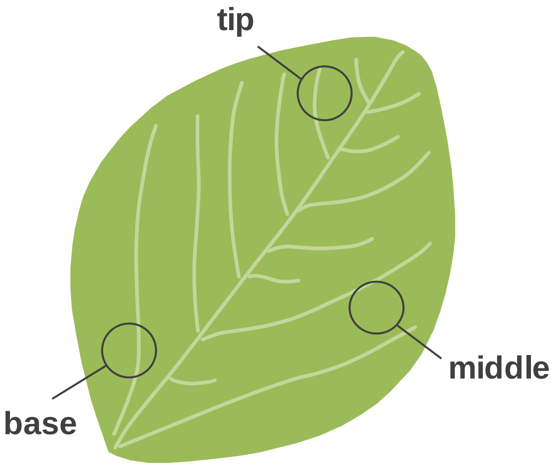

Abaxial side of the leaflet

Fig. S3 Example image showing the preparation of the training data using the image annotation tool.

In the 200 images as the training dataset, the locations of the stomata were annotated with the ground truth box using the image annotation tool, LabelImg.

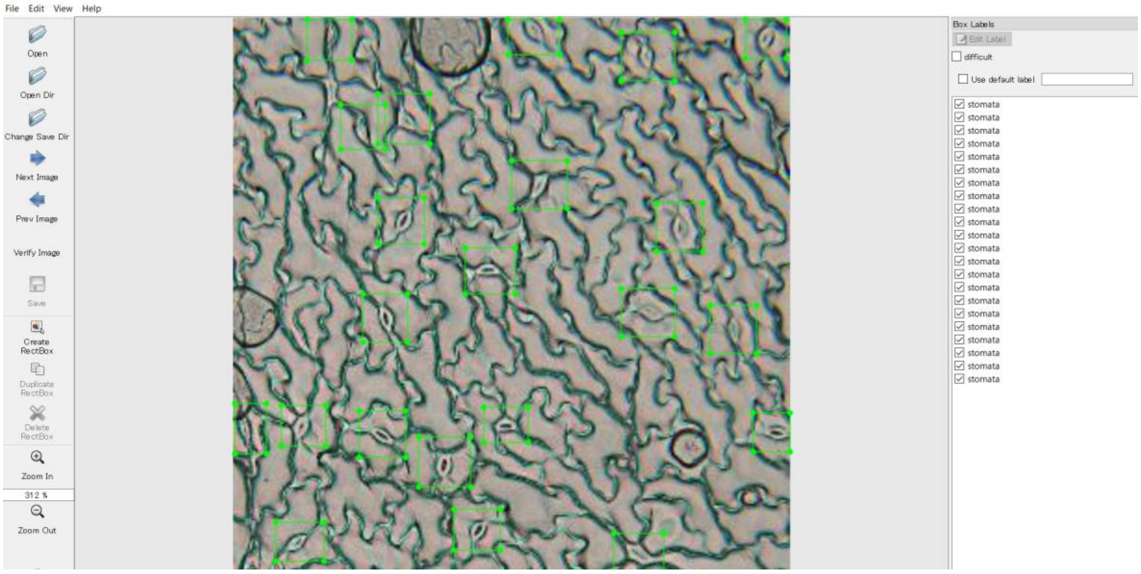

Table S1. List of line IDs, cultivar/accession names, and the origin of 90 soybean accessions.

1: The ID of each accession was assigned by the National Institute of Agrobiological Sciences (NIAS).

ID [1]	cultivar/genotype name	Origin
GmWMC001	FISKEBY V	Sweden
GmWMC006	KS 1034	Malaysia
GmWMC011	SEITA	Korea
GmWMC012	MANSHUU	China
GmWMC014	KLS 203	Korea
GmWMC015	CHUUHOKU 2	Korea
GmWMC018	RIGAI SEITOU	China
GmWMC019	CHOUSENSHU(CHA)	Korea
GmWMC020	POCHAL	Taiwan
GmWMC022	NEZUMI META	Korea
GmWMC024	CHIENEUM KONG	Korea
GmWMC027	KONGNAMUL KONG	Korea
GmWMC029	SHIROSOTA	Korea
GmWMC035	PEKIN DAI OUTOU	China
GmWMC036	MASSHOKUTOU(KOU 502)	China
GmWMC038	ICHIGUUHOU	China
GmWMC042	MASSHOKUTOU(KOU 503)	China
GmWMC045	OKJO	Korea
GmWMC046	KE 32	Philippines
GmWMC048	HEAMNAM	Korea
GmWMC066	HEUKDAELIP	Korea

GmWMC070	CHOYOUTOU	China
GmWMC071	PK 73-54	India
GmWMC072	M 581	India
GmWMC073	URONKON	Korea
GmWMC075	CHEONGYE MYONGTAE	Korea
GmWMC083	KEUMDU	Korea
GmWMC084	PEKING	China
GmWMC086	ANTO SHOUKOKUTOU	China
GmWMC089	BONGCHUNBAEKJAM	China
GmWMC094	JEOKGAK	Korea
GmWMC103	SENYOUTOU	China
GmWMC107	HAKKA ZASHI	China
GmWMC108	KARASUMAME	China
GmWMC113	BARITOU 3 A	Indonesia
GmWMC115	WILLIAMS 82	USA
GmWMC118	OUDU	Korea
GmWMC119	HAKUBI	China
GmWMC120	U 1416	Nepal
GmWMC122	GAPSANJAELAE(I)	Korea
GmWMC123	N 2295	Nepal
GmWMC125	BHATMAS	Nepal
GmWMC129	AOKI MAME	China
GmWMC132	L 2A	Philippines
GmWMC136	LOCAL VAR(SEPUTIH RAMAN)	Indonesia
GmWMC138	COL/PAK/1989/IBPGR/2326(1)	Pakistan
GmWMC141	PETEK	Indonesia
GmWMC142	JAVA 5	Indonesia
GmWMC143	M 44	India
GmWMC144	M 918	India
GmWMC146	HM 39	India

GmWMC147	COL/THAI/1986/THAI-78	Thailand
GmWMC148	M 42	India
GmWMC150	U 1042-1	Nepal
GmWMC151	JAVA 7	Indonesia
GmWMC152	U 1290-1	Nepal
GmWMC154	MANSHUU MASSHOKUTOU	China
GmWMC159	COL/PAK/1989/IBPGR/2323(2)	Pakistan
GmWMC160	N 2392	Nepal
GmWMC162	COL/THAI/1986/THAI-80	Thailand
GmWMC163	N 2491	Nepal
GmWMC166	MERAPI	Indonesia
GmWMC168	L 317	India
GmWMC169	HAKUCHIKOU	China
GmWMC170	M 652	India
GmWMC171	U-1741-2-2 NO.3	Nepal
GmWMC173	KARASUMAME(NAIHOU)	Taiwan
GmWMC175	BISHUU DAIZU	China
GmWMC176	SANDEK SIENG	Cambodia
GmWMC181	CHIENGMAI PALMETTO	Thailand
GmWMC182	LOCAL VAR.(TEGINENENG)	Indonesia
GmWMC183	KARASUMAME(HEITOU)	Taiwan
GmWMC186	RINGGIT	Indonesia
GmWMC187	KADI BHATTO	Nepal
GmWMC188	E C 112828	India
GmWMC190	SAN SAI	Thailand
GmWMC191	MISS 33 DIXI	Philippines
GmWMC192	U 1155-4	Nepal
	ENREI	Japan
not assigned	TACHINAGAHA	Japan
	STRESSLAND	USA

HOUJAKU KUWAZU	Japan
UA4805	USA
FUKUYUTAKA	Japan
ASOAOAGRI	Japan
JACK	USA
JIJORI	North korea
UA 4910	USA
JIN DOU 17	China
KOLHIDA 4	USA
